# Diet induced obesity is independent of metabolic endotoxemia and TLR4 signalling, but markedly increases hypothalamic expression of the acute phase protein, SerpinA3N

**DOI:** 10.1038/s41598-018-33928-4

**Published:** 2018-10-23

**Authors:** Matthew J. Dalby, Gabriella Aviello, Alexander W. Ross, Alan W. Walker, Perry Barrett, Peter J. Morgan

**Affiliations:** Rowett Institute, University of Aberdeen, Foresterhill, Aberdeen, AB25 2ZD United Kingdom

## Abstract

Hypothalamic inflammation is thought to contribute to obesity. One potential mechanism is via gut microbiota derived bacterial lipopolysaccharide (LPS) entering into the circulation and activation of Toll-like receptor-4. This is called metabolic endotoxemia. Another potential mechanism is systemic inflammation arising from sustained exposure to high-fat diet (HFD) over more than 12 weeks. In this study we show that mice fed HFD over 8 weeks become obese and show elevated plasma LPS binding protein, yet body weight gain and adiposity is not attenuated in mice lacking *Tlr4* or its co-receptor *Cd14*. In addition, caecal microbiota composition remained unchanged by diet. Exposure of mice to HFD over a more prolonged period (20 weeks) to drive systemic inflammation also caused obesity. RNAseq used to assess hypothalamic inflammation in these mice showed increased hypothalamic expression of *Serpina3n* and *Socs3* in response to HFD, with few other genes altered. *In situ* hybridisation confirmed increased *Serpina3n* and *Socs3* expression in the ARC and DMH at 20-weeks, but also at 8-weeks and increased SerpinA3N protein could be detected as early as 1 week on HFD. Overall these data show lack of hypothalamic inflammation in response to HFD and that metabolic endotoxemia does not link HFD to obesity.

## Introduction

Overconsumption of calories within the diet, in the absence of compensatory energy expenditure results in obesity. In part this is due to the failure of the appetite regulatory systems within the hypothalamus of the brain to detect and/or adjust to increased energy intake over the long-term. A reason for this failure may be diet itself. High-fat diets (HFD) are commonly used to induce obesity in murine and other rodent models. One theory that has emerged implicates a chronic inflammatory response in the hypothalamus, induced in rodents through feeding of a HFD, which dysregulates the neuroendocrine control of appetite and energy balance. However systemic inflammation arising from HFD can occur at different phases and via different routes. Recently it has been reported that the inflammatory response to a HFD occurs in two distinct phases; an early acute phase within a few days and a second phase after 12 to 16 weeks^1^. It is during this latter phase that inflammation from white adipose tissue and muscle also become apparent^1^. Hypothalamic inflammation resulting from long-term HFD-induced obesity was first reported in rats, with 29 immune related genes upregulated after 16 weeks feeding of a high-fat (45%) diet^2^. These genes included the cytokine tumour necrosis factor alpha (*Tnf*), interleukin 1 beta (*Il1b*), interleukin 2 (*Il2*), and interleukin 6 (*Il6*), as well as suppressor of cytokine signalling 3 (*Socs3*) from which it was suggested that local inflammation in the hypothalamus resulted in reduced sensitivity to both insulin and leptin signalling from the periphery^2^. Subsequent studies have reported that increased inflammatory gene expression can be detected in the hypothalamus of both mice and rats after only 1 to 3 days of high-fat diet feeding and prior to weight gain^3^. Furthermore, reactive gliosis and evidence of neuronal injury were apparent within the first week of HFD feeding in the hypothalamic arcuate nucleus (ARC) of rats and mice, suggesting that obesity is associated with inflammatory induced neuronal injury^3^. It has been suggested that inflammation is not simply a consequence of HFD, rather it may also drive obesity^4^.

Another potential mechanism through which diet could increase inflammation within the hypothalamus involves the gut microbiota. It has been proposed that high-fat diets alter the composition of the gut microbiota, which in turn leads to increased intestinal permeability and systemic exposure to bacterial lipopolysaccharide (LPS). This induces a state of chronic low-grade inflammation that has been called ‘metabolic endotoxemia’^5^. LPS is a highly immunogenic cell surface molecule present in the outer cell membrane of Gram-negative bacteria and it is released continuously into the gut lumen through the death of Gram-negative bacteria^6^. Under normal conditions, LPS translocates across the intestinal epithelium into the circulation, where it is neutralised and detoxified after binding to chylomicrons^7,8^. It has been proposed that high-fat diets alter the composition of the gut microbiota leading to increased numbers of Gram-negative bacteria and increased permeability of the intestinal epithelium^9^. When LPS was infused subcutaneously into mice over four weeks, to mimic the increase in LPS observed on HFD fed animals, an increase in body weight, body fat, and insulin resistance has been reported^5^. The levels of plasma LPS in response to HFD are related to the fat content of the diet and typically these are 1.4 to 2.7 times normal levels and are quite distinct from those associated with infection or septic shock, which are 10–50 times higher^10^. From these results it has been suggested that LPS can initiate obesity and insulin resistance representing a mechanism of cause and effect between the gut microbiota and body weight change^11^.

LPS is known to be a ligand for the Toll-like receptor-4 (TLR4), a transmembrane receptor on cells of the immune system^12^, although CD14 (cluster of differentiation-14) is required as a co-receptor for functional effects to be expressed^13^. Mice harbouring loss of function mutations in either *Tlr4* or *Cd14* genes have been shown to have both stronger bones and decreased body fat^14^ and this provided the first suggested link between TLR4/CD14 and adiposity. Consistent with this, CD14 deficient mice either fed a very high-fat diet (78% fat) or after low-dose LPS infusion were reported to show attenuated weight gain relative to Wild-Type mice on similar treatments^5^. Overall these results appear to demonstrate a causal link between high-fat diet feeding, the gut microbiota, LPS, and obesity and provide a basis for the metabolic endotoxemia hypothesis^11^.

While some studies have similarly reported that loss of function of either TLR4 or CD14 protects against body fat gain, insulin resistance, and inflammation in mice fed high-fat diets^15–20^ other studies have not supported such a role for CD14^21,22^ or TLR4^23–26^ in the control of body weight, while loss of TLR4 still mitigated some other negative metabolic effects of HFD feeding. The use of several different knockout mouse strains, both of TLR4 and CD14, and a variety of mouse diets has complicated our understanding of the role of the TLR4 pathway in the development of obesity. In addition, the influence of CD14 and TLR4 on body weight was not a primary aim in several of these studies.

The reasons for these contradictory results are likely to be related to differences in diet and baseline microbiota composition, nature of the loss of function (natural vs knock out) and genetic background. Therefore, in this study the aim was to compare the effects of either *Tlr4* or *Cd14* gene knock-outs in mice on the same genetic background for diet-induced obesity in response to a HFD. It also examines the long-term effects of HFD on hypothalamic inflammation. The study finds no evidence that metabolic endotoxemia contributes to HFD-induced obesity and that HFD increases the expression of only a limited number of genes in the hypothalamus yet none of these are prototypical markers of inflammation.

## Results

### Effect of diet and genotype on food intake, body weight, and body composition

**Figure 1:**
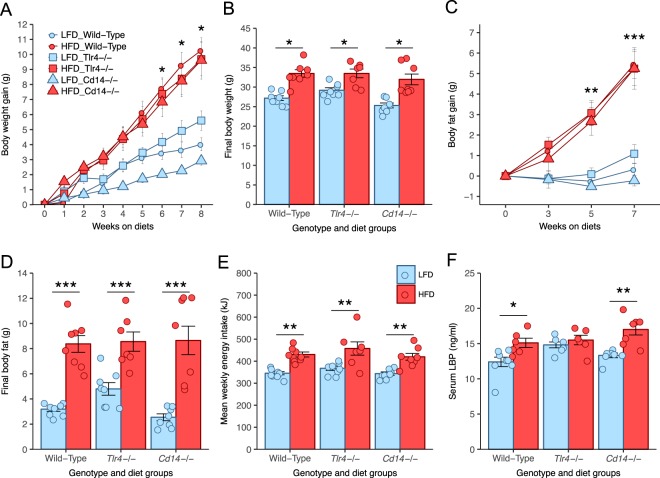
Effects of diet and genotype on body weight, body fat, energy intake, and LBP. (**A**) Weekly body weight gain; (**B**) Final total body weight; (**C**) Weekly body fat gain; (**D**) Final total body fat; (**E**) Mean weekly kilojoule intake; (**F**) Serum lipopolysaccharide binding protein (LBP); Data indicate mean ± SEM. ^∗^p < 0.05, ^∗∗^p < 0.01, ^∗∗∗^p < 0.001; n = 8 mice/group (n = 7 mice/group for HFD *Tlr4*^−/−^ mice), circles indicate individual mice.

The body weights of Wild-Type, *Tlr4*^−/−^, and *Cd14*^−/−^ mice fed HFD significantly increased relative to their respective LFD fed controls over 8 weeks (Fig. 1A). Neither the rate nor extent of body weight gain was affected by genotype. At 8 weeks the final mean group body weights of HFD fed Wild-Type, *Tlr4*^−/−^, and *Cd14*^−/−^ were significantly increased relative to their respective LFD fed controls (Fig. 1B). Mean energy intake was significantly higher for the HFD fed mice relative to their respective LFD fed controls of the same genotype (Fig. 1E). This was despite a tendency for reduced average food intake in HFD fed mice (Supplementary Fig. 1C). This resulted from the higher energy density of the HFD, which was 21.9 kJ/g compared to 16.1 kJ/g for the LFD (Supplementary Table 1). There were no significant differences in mean food or mean energy intake between mouse genotypes fed the LFD or between those fed HFD. Neither the TLR4 knockout nor the *Cd14* knockout altered food or energy intake compared to Wild-Type mice fed the HFD. Increased adiposity accounted for the increased body weight in response to HFD by each genotype (Fig. 1C), with a significant accumulation of body fat in each of the HFD groups relative to their respective controls (Fig. 1C,D). By contrast there were no differences in the lean mass of any of the experimental groups at any time point (Supplementary Fig. 1D–E). Overall neither the *Tlr4* nor the *Cd14* knockout protected mice from the HFD induced increases in body fat.

### Effect of high-fat diet and genotype on lipopolysaccharide binding protein concentrations

Serum lipopolysaccharide binding protein (LBP) concentrations were measured as a marker of systemic lipopolysaccharide exposure (Fig. 1F). In response to HFD feeding, serum concentrations of LPB in Wild-Type and *Cd14*^−/−^ mice were each significantly increased relative to the LFD controls of their corresponding genotype. In contrast there was no significant difference in serum LPB concentrations between the LFD and HFD fed TLR4^−/−^ mice. Serum LPB concentrations in HFD fed Wild-Type, *Tlr4*^−/−^, and *Cd14*^−/−^ mice were all significantly higher than in Low-Fat diet fed Wild-Type control mice. High-Fat diet feeding increased serum LPB concentrations, suggesting increased LPS exposure.

### High fat diet and genotype and the gut microbial composition

**Figure 2:**
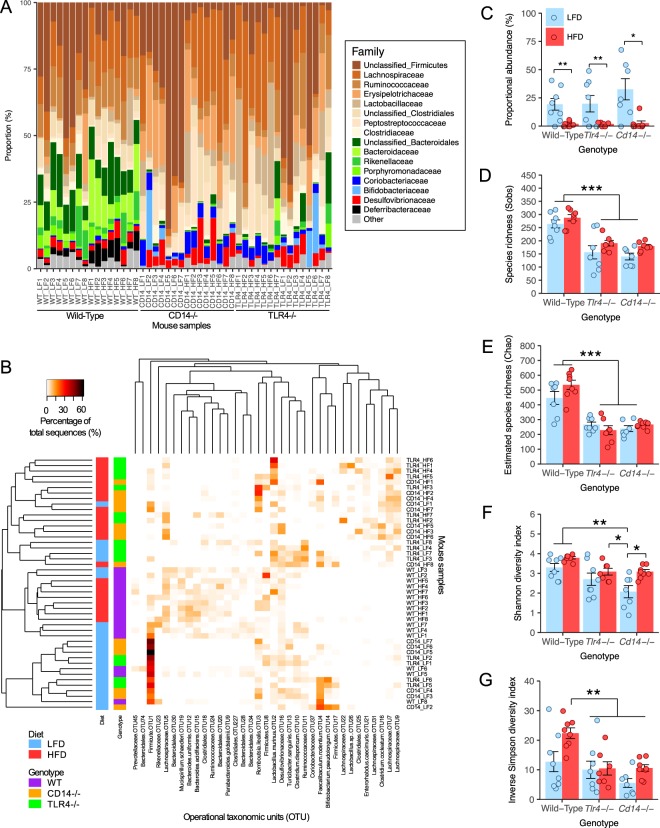
Effects of diet and genotype on caecal microbiota composition. (**A**) Proportional abundance of bacterial families in Wild-Type, *Tlr4*^−/−^, and *Cd14*^−/−^ mice fed LFD or HFD with Firmicutes phylum in browns, Bacteroidetes in greens, Actinobacteria in blues, and Deferribacteres in black; (**B**) Proportional abundance of bacterial OTUs in Wild-Type, *Tlr4*^−/−^, and *Cd14*^−/−^ mice fed LFD or HFD. Heatmap of proportion of OTUs (>3% proportional abundance) in the caecum, with rows clustered by microbiota similarity using the Bray-Curtis calculator, and columns clustered by OTUs that occur more often together; (**C**) Proportional abundance of OTU1; (**D**) Species richness (Sobs); (**E**) Estimated species richness (Chao); (**F**) Shannon diversity index; (**G**) Inverse Simpson diversity index. Data are mean ± SEM. n = 8 mice/group (n = 7 mice/group for HFD *Tlr4*^−/−^ and LFD *Cd14*^−/−^ mice), circles indicate individual mice. ^∗^p < 0.05, ^∗∗^p < 0.01, ^∗∗∗^p < 0.001.

Illumina MiSeq sequencing of the V1-V2 variable region of the 16S ribosomal RNA gene was used to analyse the composition of the caecal microbiota of Wild-Type, *Tlr4*^−/−^, and *Cd14*^−/−^ mice after 8 weeks of diet feeding. Comparing the proportional abundance of bacterial phyla and families between groups shows clear differences between the Wild-Type and both *Tlr4*^−/−^ and *Cd14*^−/−^ mice (Fig. 2A). The proportion of Bacteroidetes was higher in Wild-Type mice, compared to *Tlr4*^−/−^ and *Cd14*^−/−^ mice. The proportional abundance of Firmicutes was significantly lower in LFD than HFD fed Wild-Type mice and lower in all Wild-Type mice compared to all *Tlr4*^−/−^ and *Cd14*^−/−^ mice (Supplementary Fig. 3A). Bacteroidetes, and Proteobacteria were not significantly changed between HFD and LFD fed mice within each genotype (Supplementary Fig. 3B,C). The proportion of Actinobacteria was lower in Wild-Type than *Tlr4*^−/−^ and *Cd14*^−/−^ mice (Supplementary Fig. 3D). The proportion of Deferribacteres was higher in HFD Wild-Type mice relative to LFD fed Wild-Type mice but was not detected in either the *Tlr4*^−/−^ and *Cd14*^−/−^ mice (Supplementary Fig. 3E). There were no differences in the proportions of Verrucomicrobia (Supplementary Fig. 3F). The influence of diet on microbiota composition at the phylum level was not shared across mouse genotype. The proportional abundance of Gram-negative bacteria was significantly lower in *Tlr4*^−/−^ and *Cd14*^−/−^ mice compared to Wild-Type mice but was unchanged between HFD and LFD fed mice in each genotype (Supplementary Fig. 3G).

Dendrograms based on the Bray-Curtis dissimilarity index show that the microbiota of Wild-Type mice cluster distinctly from both *Tlr4*^−/−^ and *Cd14*^−/−^ mice (Fig. 2B). The microbiota of Wild-Type mice fed LFD and HFD clustered closely together and comprised primarily of members of the Firmicutes and Bacteroidetes phyla. Strikingly, relatively few differences in the proportion of the bacterial Families could be seen between the LFD and HFD groups, irrespective of genotype. In contrast, the microbiota of the *Tlr4*^−/−^ and *Cd14*^−/−^ mice were composed primarily of Firmicutes in both diets. No clear distinction was observed in the clustering of *Tlr4*^−/−^ and *Cd14*^−/−^ mice within each diet group. The *Tlr4*^−/−^ and *Cd14*^−/−^ mice contained a different microbial composition that was altered to a greater degree in response to diet than the Wild-Type mice (Fig. 2B). The difference in microbiota between LFD fed *Tlr4*^−/−^ and *Cd14*^−/−^ mice was primarily due to the greater abundance of an unclassified Firmicutes (OTU1) in LFD fed mice of all genotypes (Fig. 2C). The number of Operational Taxonomic Units (OTUs) (Sobs) and estimated total OTU richness (Chao) were lower in *Tlr4*^−/−^ and *Cd14*^−/−^ mice compared to Wild-Type mice, while there were no differences between LFD and HFD fed mice within each genotype (Fig. 2D–E).

The microbiota diversity of the caecum was measured using the Shannon and Inverse Simpson indices, which incorporate species richness and evenness of the community when calculating diversity scores. Shannon and Inverse Simpson indices tended to be decreased in the caecal microbiota of *Tlr4*^−/−^ and *Cd14*^−/−^ mice compared to Wild-Type mice (Fig. 2F–G). The *Tlr4*^−/−^ and *Cd14*^−/−^ mice contained a different microbiota composition to Wild-Type mice. Differences in OTU richness as measured by Sobs and Chao were between Wild-Type mice and *Tlr4*^−/−^ and *Cd14*^−/−^ mice. However, there were no consistent differences in Shannon and Inverse Simpson indexes between diets groups or by genotype.

### Gene expression of *Tlr2*, *Tlr4* and *Cd14* in the hypothalamus

**Figure 3:**
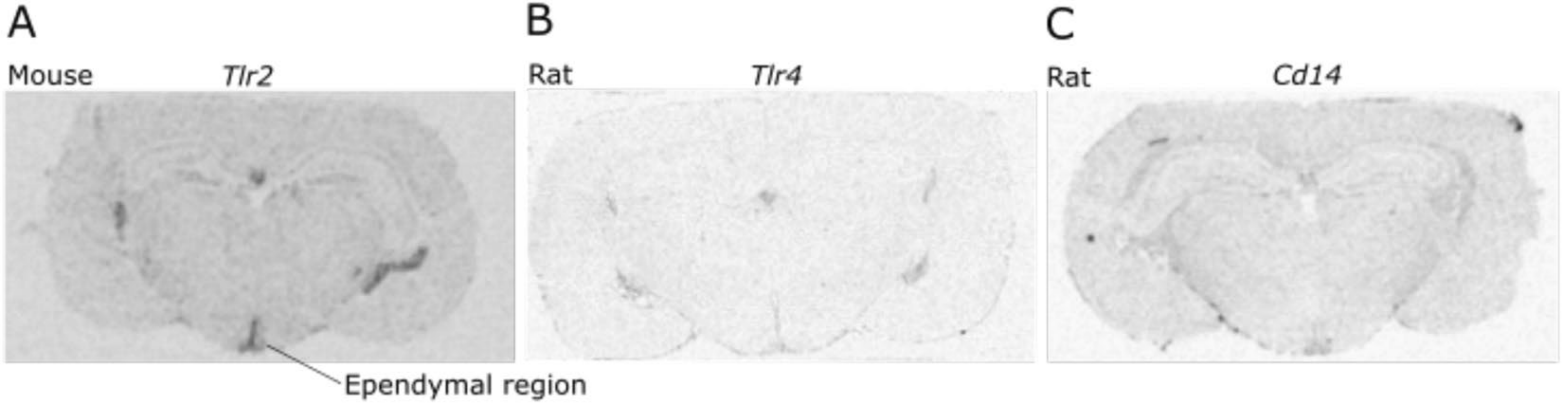
*Tlr2* and *Tlr4* gene expression in the hypothalamus of the mouse and rat. *Tlr2* gene expression in ependymal cells/tanycytes (hypothalamo-ventricular zone) of mouse (**A**). Very weak expression of Tlr4 and CD14 is detectable in the HVZ of the rat (**B**,**C**).

Expression of *Tlr2*, *Tlr4*, and *Cd14* genes in the hypothalamus was assessed by *in situ* hybridisation in the mouse and rat (Fig. 3). Sections from rat brains were used to test probe function due to the difficulty in detecting *Tlr4*, and *Cd14* gene expression in mouse brain sections. While expression of *Tlr2* could be detected in the hypothalamo ventricular zone (HVZ) of the mouse, expression of both *Tlr4* and *Cd14* was undetectable. Very weak expression of *Tlr4* and *Cd14* was detectable in the hypothalamus of the rat, mainly associated with the HVZ. These findings indicate that any effects of LPS through TLR4 or CD14 are unlikely to be through direct action within the hypothalamus.

### Effect of high fat diet on hypothalamic gene expression

**Figure 4:**
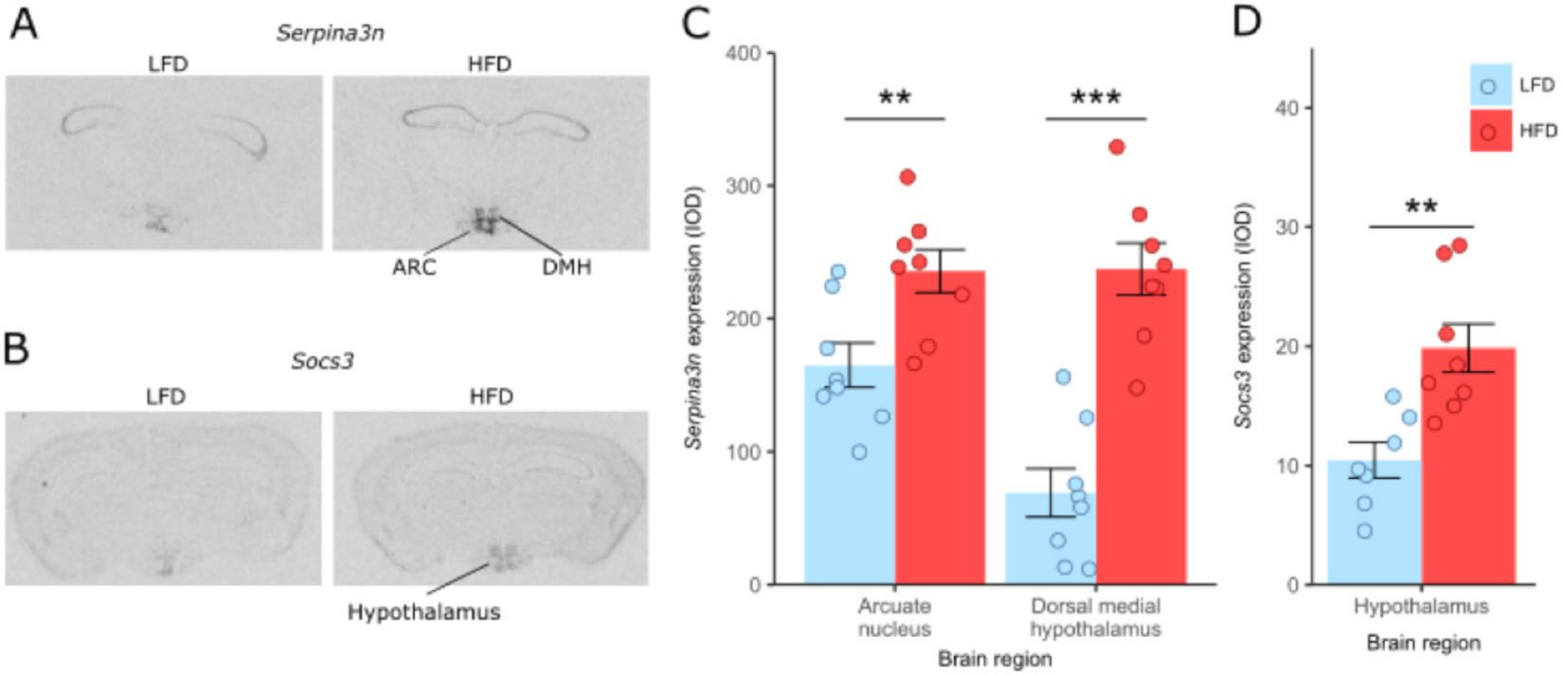
Effects of 20 weeks of LFD and HFD on hypothalamic *Serpina3n* and *Socs3* expression. (**A**) *In situ* hybridization expression of *Serpina3n* in the hypothalamus of LFD and HFD fed Wild-Type mice; (**B**) *In situ* hybridization expression of *Socs3* in the hypothalamus of LFD and HFD fed Wild-Type mice; (**C**) *In situ* hybridization of *Serpina3n* expression in the arcuate nucleus (ARC) and dorsal medial hypothalamus (DMH) of Wild-Type mice; (**D**) *In situ* hybridization of *Socs3* expression in the hypothalamus of Wild-type; n = 7–8 mice/group; Data are mean ± SEM, circles indicate individual mice. ^∗^p < 0.05, ^∗∗^p < 0.01, ^∗∗∗^p < 0.001.

To capture the effect of a sustained HFD-induced inflammatory response, RNAseq was used to analyse differential gene expression within the hypothalamus in mice fed either LFD or HFD for 20 weeks (Supplementary Fig. 4). From an initial 46095 reads, 16841 genes were expressed sufficiently to permit analysis. Of these only 6 genes had significantly different expression between the two diets at a false discovery rate (FDR) ≤ 0.05 (Table 1). All six genes were more highly expressed in mice on a HFD relative to a LFD and they have a mixture of functions including serine peptidase inhibitor A3N (*Serpina3n*), transcriptional and intracellular signalling including both the signal transducer and activator of transcription 3 (*Stat3*) and suppressor of cytokine signalling 3 (*Socs3*) or as pseudogenes with no known functional protein coding capacity. Differential expression of *Serpina3n* and *Socs3* after 20 weeks of high fat relative to low fat diet was confirmed by *in situ* hybridisation (Fig. 4).

**Table 1:** Genes with significantly increased hypothalamic expression between mice fed high fat diets relative to low fat fed controls, identified by edgeR (FDR < 0.05).

Gene Name	Fold change	Log fold change	P-value	FDR	Gene description*
*Serpina3n*	**1**.**7**	−0.793236193	5.06E-09	8.52E-05	Serine (or cysteine) peptidase inhibitor
LOC10524359	**62**.**8**	−5.972717972	1.87E-07	0.001571933	60S ribosomal protein L23a pseudogene
*Stat3*	**1**.**2**	−0.266379652	1.01E-06	0.005687007	Signal transducer and activator of transcription 3
*Gm6210*	**14**.**5**	−3.86002715	3.88E-06	0.016325199	Heat shock protein 8 pseudogene
*Socs3*	**1**.**6**	−0.655744451	9.62E-06	0.032403367	Suppressor of cytokine signalling 3
GM15459	**8**.**7**	−3.112752289	1.44E-05	0.040307169	Heat shock protein 8 pseudogene

^*^As given by NCBI Gene Database.

Differential expression of 2 of the known genes, *Serpina3n* and *Socs3*, was verified using *in situ* hybridisation (Fig. 4). *Serpina3n* gene is expressed in the arcuate (ARC) and dorsomedial (DMH) nucleus regions of the hypothalamus. After 20 weeks of HFD, *Serpina3n* gene expression was significantly increased in both regions compared to mice on LFD, although the magnitude of the change is greatest in the DMH (Fig. 4A,C). Similarly, *Socs3* gene expression within the hypothalamus also increased by 20 weeks of HFD (Fig. 4B,D).

**Figure 5:**
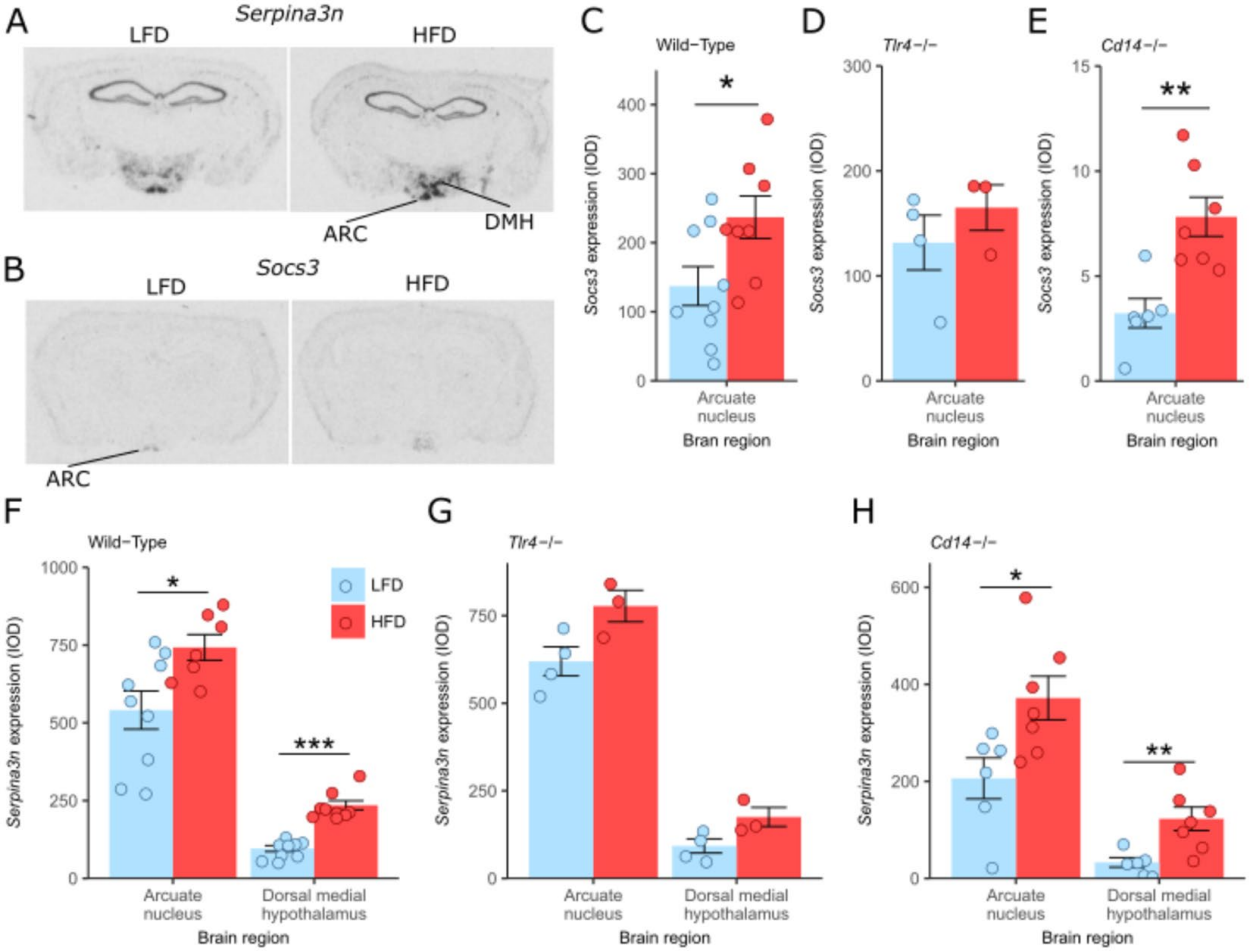
Effects of 8 weeks of diet and genotype on hypothalamic *Serpina3n* and *Socs3* expression. (**A**) *In situ* hybridization expression of *Serpina3n* in the hypothalamus of LFD and HFD fed Wild-Type mice at 3 days exposure; (**B**) *In situ* hybridization expression of *Socs3* in the hypothalamus of LFD and HFD fed Wild-Type mice at 7 days exposure; (**C**) *In situ* hybridization of *Socs3* expression in the arcuate nucleus (ARC) of Wild-Type mice, (D) *Tlr4*^−/−^ mice, and (**E**) *Cd14*^−/−^ mice; (**F**) *In situ* hybridization of *Serpina3n* expression in the arcuate nucleus and dorsal medial hypothalamus of Wild-Type, (**G**) *Tlr4*^−/−^, and (**H**) *Cd14*^−/−^ mice; n = 6–9 mice/group (n = 3–4 mice/group for *Tlr4*^−/−^); Data are mean ± SEM, circles indicate individual mice. ^∗^p < 0.05, ^∗∗^p < 0.01, ^∗∗∗^p < 0.001.

Using *in situ* hybridisation, gene expression for *Serpina3n* and *Socs3* was then assessed in brains from Wild-Type, *Tlr4*^−/−^, and *Cd14*^−/−^ mice fed HFD or LFD for only 8 weeks (Fig. 5). This showed that gene expression of *Serpina3n* and *Socs3* were also both increased in the hypothalamus by HFD relative to LFD after 8 weeks of dietary treatment, implying that these are early phase as well as second phase responses. The pattern of expression was similar to the changes seen at 20 weeks (Fig. 5A-B). For both Wild-Type and *Cd14*^−/−^ mice *Serpina3n* was significantly increased in both the ARC and dorsal medial hypothalamus (DMH), although the magnitude of the increase was greater in the DMH (Fig. 5F,H). Also, the baseline level of expression for *Serpina3n* was greater in the ARC at 8 weeks relative to 20 weeks (Figs 4 and 5). There were similar increases in *Serpina3n* expression in the ARC (P = 0.051) and the DMH (P = 0.054) between the HFD and LFD in the TLR4^−/−^ mice (Fig. 5G). *Socs3* gene expression in the hypothalamus of Wild-Type and *Cd14*^−/−^ mice was also significantly increased after 8 weeks HFD dietary treatment relative to LFD controls (Fig. 5C,E), although no difference in expression for this gene was observed for *Tlr4*^−/−^ mice (P = 0.38) (Fig. 5D).

**Figure 6:**
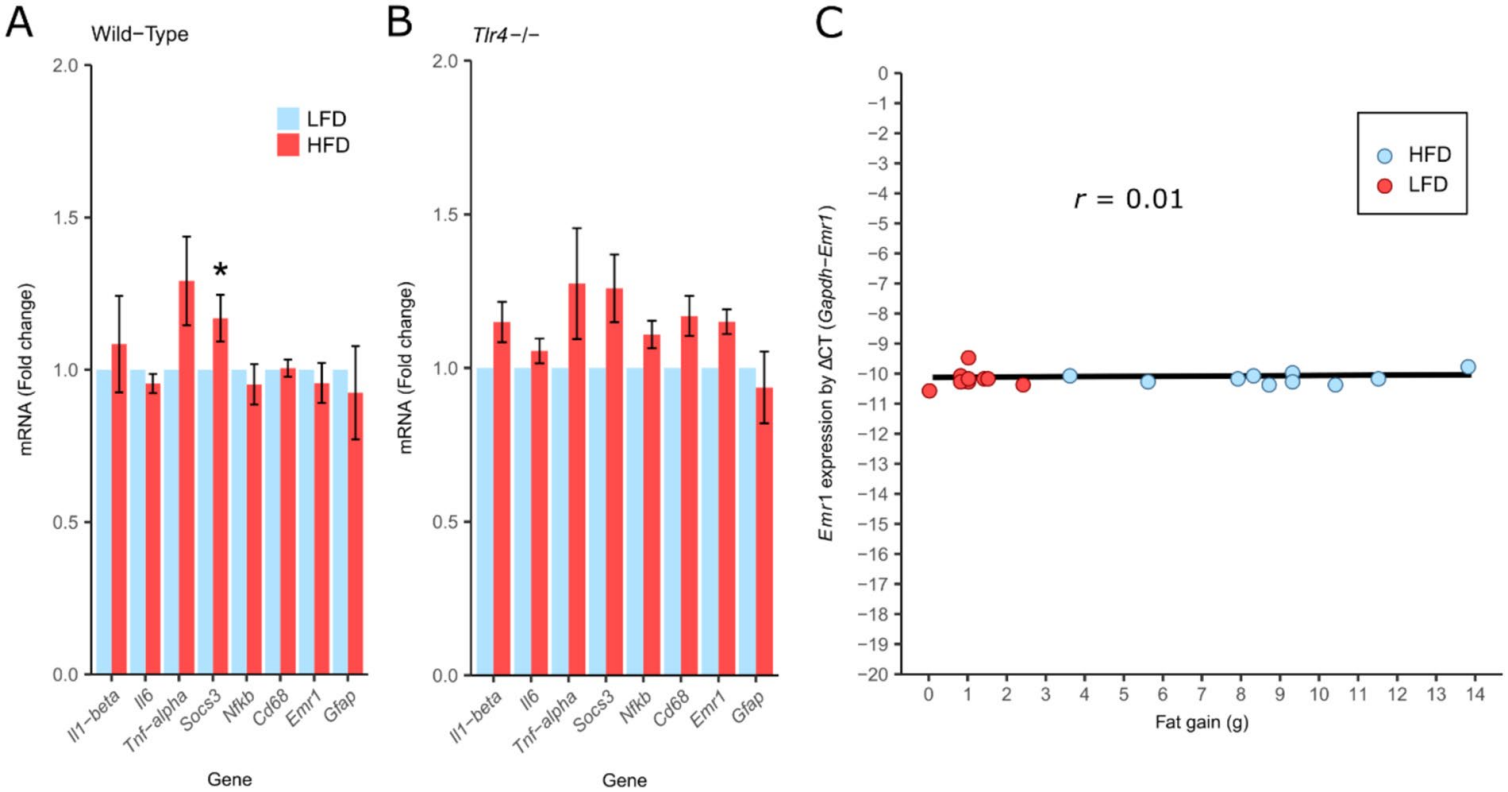
Effects of diet and *Tlr4*^−/−^ genotype on hypothalamic inflammatory gene expression. (**A**) Fold change in hypothalamic genes in response to high-fat diet in Wild-Type mice; (**B**) Fold change in hypothalamic genes in response to high-fat diet in *Tlr4*^−/−^ mice; (**C**) Correlation of hypothalamic *Emr1* mRNA level (linearized using difference in CT count between the *Gapdh* and the *Emr1* gene [ΔCT]) with change in fat mass in Wild-Type mice fed both LFD and HFD (*r* = 0.01; p = 0.6). mRNA was quantified relative to *Gapdh* housekeeping gene expression (by ΔΔCT method) and presented as fold change relative to LFD-fed controls; Significant differences were determined using REST software^66^; ^∗^p < 0.05. Values are expressed as the mean ± standard error; n = 6 for Wild-Type mice and n = 4 for *Tlr4*^−/−^ mice.

As other studies have previously reported increased expression of other genes have been associated with hypothalamic inflammation in mice fed a HFD after 28 days^3^, Real-Time PCR was used to assess their expression (Fig. 6). These data show that of the genes tested *Socs3* was significantly increased in the hypothalamus of Wild-Type mice fed HFD for 8 weeks, but not in TLR4^−/−^ mice relative to their respective LFD controls, consistent with *in situ* hybridisation results (Fig. 6A,B). None of the other inflammatory-related genes tested, *Il1*, *Il-6*, *Tnf* *Nfκb*, *Cd68*, *Gfap* and *Emr1* had altered expression in response to HFD (Fig. 6A,B). Expression of the myeloid cell-specific marker *Emr1*, which has previously been correlated with body fat gain^3^, showed no such correlation (Fig. 6C). Furthermore, the low normalised delta CT values determined using Real Time PCR for Il1b, Il6 and Tnf indicate that the expression of these inflammatory genes was very low in the hypothalamus (Supplementary Fig. 2).

**Figure 7:**
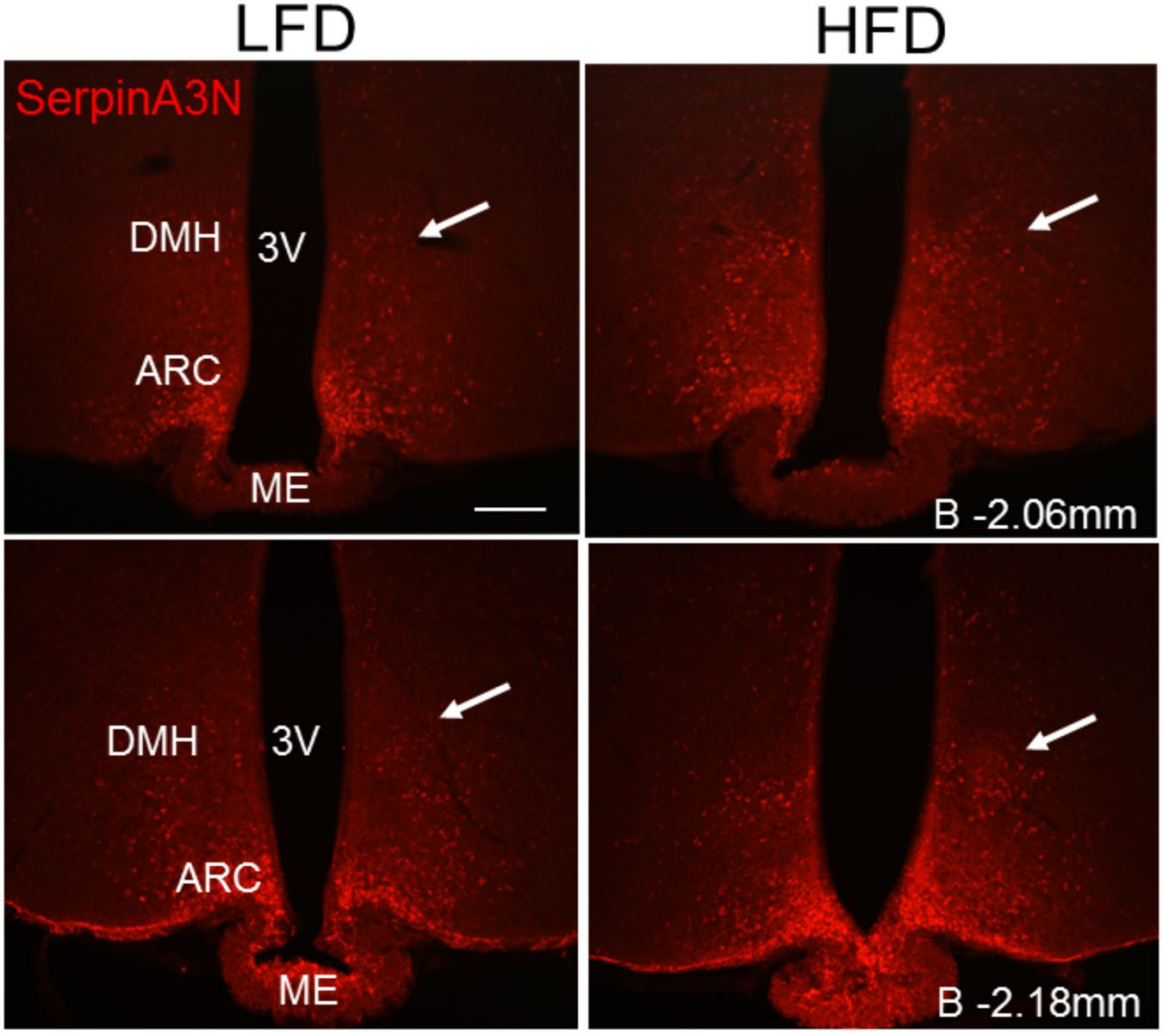
Effects of diet on hypothalamic SerpinA3N protein expression. SerpinA3N (AF-594 conjugated) expression in the ARC and DMH of Wild-Type mice fed with LFD and HFD for one week. Images were captured at two different bregma levels and are representative of n = 5 per group. ME: median eminence; 3: third ventricle; B: bregma; ARC: arcuate nucleus; DMH: dorsomedial hypothalamus. Scale bar = 100 μm.

Given the finding that *Serpina3n* gene expression is robustly increased in the hypothalamus in response to HFD at both 8 and 20 weeks, we sought to examine the expression of SerpinA3N protein in the hypothalamus. Immunoreactive SerpinA3N could be detected in the ARC of Wild-Type mice fed LFD (Fig. 7). Remarkably after only 1 week of feeding mice a HFD, increased expression of SerpinA3N immunoreactive protein in the DMH could be observed (see arrows Fig. 7), with little change in immunoreactivity in the ARC (Fig. 7). These data indicate that increased expression of SerpinA3N is an early and sustained response to HFD. The absence of co-localization in the double staining with GFAP showed that SerpinA3N is not expressed by astrocytes in the hypothalamus (Supplementary Fig. 5). Instead the shape and location of the immunoreactive staining suggests that SerpinA3N is expressed in hypothalamic neurons.

## Discussion

In this study whole-body knockout of either *Tlr4* or *Cd14* in mice did not protect against increased body weight, body fat, or energy intake relative to Wild-Type mice, when fed HFD over an 8-week period. Despite this, LBP, a biomarker for LPS exposure^27–30^ increased in response to HFD in both Wild-Type and *Cd14*^−/−^ mice, suggesting that mice fed a HFD were exposed to higher levels of LPS than LFD mice. Together these data argue that LPS, which acts via the *TLR4*/CD14 receptor complex, does not play a role in HFD-induced obesity. This data contradicts two studies in the literature reporting that *Cd14*^−/−^ mice^5,15^, and *ob/ob Cd14*^−/−^ double knockout mice^11^, are protected from high-fat diet induced body fat gain whilst consistent with two studies which have reported increased body weight or body fat in HFD fed *Cd14*^−/−^ mice^21,22^.

Thus, our results do not support the concept of metabolic endotoxemia and its role in the development of obesity, which stemmed from studies showing that Wild-Type mice fed a HFD have chronically elevated levels of LPS^5^. In these studies chronic infusion with low doses of LPS was shown to mimic the effects of HFD-induced obesity in mice. Crucially, the effect of HFD on weight gain and other metabolic parameters were attenuated in knockout mice lacking CD14, the co-receptor required for the biological activity of LPS^5^, but our results contrast starkly with these.

That *Tlr4*^−/−^ mice also were not protected against HFD-induced obesity in this study, is consistent with the results for *Cd14*^−/−^ mice, since the latter is a required co-receptor^13^. However, the effects of *Tlr4* knock-outs on HFD-induced obesity reported in the literature are contradictory^15–17,19,20,22–26^. There is no clear explanation for the diverse responses that exist, but it is likely that the strain and sex of the mice used, the nature of the knockout (naturally occurring mutation, deletion, or targeted gene knock out), variance in microbiota composition and activities, and the type of high-fat diet used are all relevant factors. The current study is the first to directly test the effect of both *Tlr4* and *Cd14* gene knock-outs on HFD-induced obesity in the same study. The consistency of the findings strongly supports the conclusion that there is no effect of the TLR4/CD14 receptor complex on weight gain, body fat, or food intake in mice.

In addition to acting as a co-receptor for TLR4, CD14 also acts as a co-receptor for Toll-like receptor 2 (TLR2). CD14 is required for TLR2 responses to pathogen infection^31,32^, and is required for bacterial product-induced anorexia resulting from TLR2 ligands^33^. The loss of TLR2 has previously been reported to reduce high-fat diet-induced increase in body fat in mice^34,35^, while other evidence suggests functional hypothalamic neuronal TLR2 protects against age-induced obesity^36^. The lack of any measurable differences in body weight or body fat gain seen in *Cd14*^−/−^ mice, compared to Wild-Type or *Tlr4*^−/−^ mice, suggests that TLR2 signalling is unlikely to have a role in diet-induced obesity.

While the current study finds no evidence for a role of TLR4 /CD14 in HFD-induced obesity, the increase in LPB in response to HFD in Wild-Type and *Cd14*^−/−^ mice is indicative of increased LPS exposure. This is consistent with previous observations that HFD increases circulating levels of LPS^5^. It has been postulated that a potential mechanism involved in this process is a change in gut microbial composition^9^. In the present study using 16S rRNA gene sequencing there were relatively few differences in the composition of the caecal microbiota between mice on low-fat and high-fat diets, within each genotype. There was no difference in the proportional abundance of the Firmicutes and Bacteroidetes phyla due to diet, or in the ratio between them. This contrasts with earlier reports of an obesity associated microbiota^37^, but is consistent with our recent study which shows the importance of rigorous dietary control when assessing gut microbial composition^38^. This is because, if chow diets are used as controls for defined high fat diets, then the different levels of fibre between the two diets leads to misleading microbiota analysis and interpretation^38^. However, there are other ways in which the microbiota might be playing a role, which would not be detected using solely 16S rRNA gene sequencing. As the LFD was high in digestible starch and low in fibre this could reduce bacterial fermentation and short-chain fatty acid (SCFA) synthesis, which has been suggested to result in thinning of the gut mucus layer potentially increasing bacterial translocation and triggering inflammation^39^. While caecal SCFA were not determined in this study the same diets previously resulted in no differences in caecal SCFA concentrations^38^. In addition HFD feeding could potentially increase the proportion of Gram-negative bacteria in the gut increasing LPS exposure. However, in this study the proportion of Gram-negative bacteria was unchanged between in HFD fed mice compared to their respective genotype controls.

There were however, marked differences between the microbiota composition and diversity of both the *Tlr4*^−/−^ and *Cd14*^−/−^ mice in comparison to the Wild-Type mice. The loss of TLR4 signalling potentially influences microbiota composition as the TLR4 is one of the pattern recognition receptors through which bacteria in the gut shape the host’s immune tolerance and responsiveness. However, homozygous TLR4 knockout mice do not have significantly different caecal microbiota composition relative to heterozygous littermates, suggesting that familial transmission is more important than genotype^40^. Recent research indicates that the intestinal microbiota composition of mice varies between different facilities^41^ and even between mice maintained in different rooms within the same animal facility^42^ and this could be a potential explanation for the observed differences in microbiota composition.

Overall Wild-Type mice did not show an obesity associated microbiota despite large increases in body fat in high-fat fed Wild-Type mice. This is consistent with our previous findings^38^ and re-confirms that change in microbiota composition is not a prerequisite for obesity development. The major differences in gut microbial composition of the *Tlr4*^−/−^ and *Cd14*^−/−^ mice did not lead to differences in susceptibility to obesity compared to the Wild-Type mice. Thus, these results suggest that the development of HFD-induced obesity in mice can be disassociated from changes in the composition of the caecal microbiota and is therefore unlikely to have contributed to differences in circulating LPS.

While this study shows that neither *Tlr4* and *Cd14* have a role in the HFD-induced obesity HFD has the potential to influence obesity development through *Tlr4* and *Cd14* independent pathways. Thus other systemic inflammatory responses associated with HFD provide alternative ways of driving hypothalamic inflammation^2,3^. This inflammation has also been linked to hypothalamic resistance to insulin and leptin signalling, potentially contributing to energy imbalance and obesity^43^.

Given that the inflammatory response to HFD occurs in two distinct phases, an acute short term response (days) and a longer-term response (>12–16 weeks)^1^, RNAseq was carried out on hypothalamic tissue from mice fed either LFD or HFD for 20-weeks, to capture the long-term effects of systemic inflammation on the hypothalamic response. Suprisingly, few gene expression changes were observed, contrasting to a previous report in Wistar rats where 29 immune-related genes were upregulated, including *Tnfα*, *Il1β*, and *Il6* after exposure to HFD for 16 weeks^2^. Of the selected inflammatory genes reported as upregulated in rats in response to high-fat diet induced obesity^3^, only *Socs3* expression was significantly altered in the present study. This response to HFD was seen at both 8 and 20-week time points indicating that it is both a short-term as well as long term response to HFD. Such increased expression of *Socs3* in the hypothalamus is likely to reflect increased resistance to insulin and leptin signalling, which is known to occur in HFD-induced obesity^44^. The increase in *Socs3* expression in *Cd14*^−/−^ mice also shows that TLR4 signalling is not required for this response.

A striking finding from these studies is that expression of the gene encoding the acute-phase protein SerpinA3N is robustly up-regulated in the ARC and DMH of mice fed HFD at both 8 and 20 weeks relative to LFD fed controls. *Serpina3n* encodes the protein alpha 1-antichymotrypsin, which is mainly produced in the liver and involved in the resolution of inflammation and wound healing^45^. In the brain SerpinA3N has been reported to be a marker of reactive astrogliosis^46^, however in this study its expression was not associated with astrocytes. SerpinA3N has also recently been shown to induce neuroprotection both *in vitro* and in mouse models of neurodegenerative disease^47^ and increased *Serpina3n* gene expression was the only gene found to be upregulated in the hypothalamus of mice in another recent study of the response to HFD feeding^48^. Interestingly, immunofluorescence staining showing increased SerpinA3N protein expression can be observed after just one week of high fat diet feeding, consistent with early responses to HFD in the hypothalamus seen in other studies^49^. This shows that increased *Serpina3n* expression in the hypothalamus is both a rapid as well as sustained response to HFD.

Overall these results show that changes in hypothalamic gene expression in response to 8 and 20 weeks of high-fat diet feeding were limited to a small number of genes but none of these are prototypical inflammatory or immune responses. The increase in *Serpina3n* mRNA expression suggests a response to undetected inflammation or neuronal damage, whereas the increase in *Socs3* mRNA expression probably reflects increased insulin and leptin resistance in the hypothalamus. Hypothalamic endoplasmic reticulum stress due to increased ceramide, a lipotoxic metabolite of fatty acid metabolism, is an alternative pathway of hypothalamic damage in high-fat diet fed mice^50,51^. As neither the *Serpina3n* nor *Socs3* responses were abrogated in *Cd14* knockout mice, then neither of these responses involve TLR4 and therefore are not induced by LPS.

In conclusion these results show susceptibility to a high fat diet is independent of TLR4 signalling and do not support the concept of metabolic endotoxemia as a determinant of obesity. We also conclude that caecal microbiota composition or diversity is not consistently altered within genotypes as a result of consuming a high fat diet. This data therefore does not support the notion that changes in the proportional composition of the gut microbiota underpin changes in susceptibility to obesity. In addition we did not observe an inflammatory response in the hypothalamus to HFD-induced obesity as seen in other studies^1,2^. Instead the striking response was a robust increase in the acute-phase protein SerpinA3N which was sustained throughout the period of HFD treatment. This provides new evidence about the pathophysiological effects of HFD-induced obesity within the neuroendocrine hypothalamus, potentially arising from fatty acid metabolism. These results have potentially broad significance to our understanding of the role of diet in the development of obesity in animals as well as humans.

## Methods

### Ethics statement

All animal experiments were performed under strict adherence to UK Home Office regulations according to the Animals (Scientific Procedures) Act, 1986, and were licensed by the UK Home Office under Project License PPL60/4282 and approved by the local ethics committee at the Rowett Institute and University of Aberdeen (Approval number: SA13/03E).

### Experimental animals

#### Mice used in the 8 week feeding experiment

Male mice at 6–7 weeks of age including; 16 Toll-like receptor-4-null (TLR4^−/−^) mice (B6.B10ScN-Tlr4lps-del/JthJ, Stock Number 007227), and 16 Cluster of Differentiation-14-null (CD14^−/−^) mice (B6.129S4-Cd14tm1Frm/J, Stock Number 003726) were purchased from the Jackson Laboratory (Bar Harbor, ME, USA). Wild-Type C57BL/6J (JAX™ Mice Strain, Stock Number 000664) control mice at 6–7 weeks of age were obtained from a UK based breeding colony of JAX® Mice maintained by Charles River (Charles River, UK). All mice were housed under specific pathogen free conditions in the Medical Research Facility at the University of Aberdeen. The room was maintained at 21 ± 2 °C and 55 ± 10% relative humidity with a standard 12 h light, 12 h dark lighting regime with lights on from 7 am to 7 pm. Experimental diets were the HFD D12492 (60 kcal% fat) (Research Diets, Inc.) and the LFD control D12450B (10 kcal% fat) (Research Diets, Inc.) matched for sucrose content, (Supplementary Table 1). Mice were offered food and water *ad libitum* throughout the study.

On arrival, the mice were acclimatised for 2 weeks in group housed cages of 8 or 10 mice per cage and with *ad libitum* commercial mouse chow (Special Diet Services, Witham, UK). Sixteen mice of each genotype, Wild-Type, *Cd14*^−/−^, and *Tlr4*^−/−^, were switched to singly housed shoebox cages containing a grid floor and food intake recording started. All mice were then switched onto the LFD for 1 week. Wild-Type control mice not requiring food intake measurements were singly housed in solid floored cages containing sawdust bedding. For increased enrichment of the cage environment all cages contained a roof-suspended plastic mouse house, a plastic tunnel, and shredded paper for bedding and nesting. After one week on the LFD, the mice were weighed and randomised into weight matched diet groups. One half of the mice of each genotype were switched to the HFD, while the remaining mice continued the LFD for 8 weeks.

Food intake was weighed and recorded twice weekly on Monday and Thursday by weighing food added to the hopper and weighing uneaten food on the next measurement day. At the same time, spilt food was collected from the trays under the grid floor cages and weighed and deducted from the food eaten. Bodyweights for all mice were recorded weekly. Whole body composition (as total fat and lean masses) was quantified at the beginning of the study and from then on, every 2 weeks by magnetic resonance imaging (MRI; EchoMRI, 2004, Echo Medical Systems, Houston, TX, USA) which measures the mass of fat and lean tissue in live animals using nuclear magnetic resonance (NMR) technology. The EchoMRI machine was calibrated using canola oil as a reference fat.

The study was completed after 8 weeks of feeding the experimental diets. Mice were killed by cervical dislocation. Trunk blood was collected via decapitation. The brains were removed, snap frozen on dry ice and then stored at −80 °C. The caecum contents were collected, frozen on dry ice and then stored at −80 °C.

#### Mice used in the 20 week feeding experiment

Thirty-two male C57BL/6J mice were purchased at 3–4 weeks old from Harlan UK. Mice were acclimatized for two weeks. During the first week of acclimatization, mice were fed chow and during the second week, were fed the 10% LFD. Mice were divided into 4 body weight matched groups of 8. Two groups were fed the LFD and two groups fed the HFD for 20 weeks. Fat and lean tissue content of mice was determined at 4 weekly intervals using Echo MRI. After 20 weeks on LFD or HFD, mice were killed by cervical dislocation. The brain was removed from one group of LFD and one group of HFD fed mice and hypothalamic tissue punches prepared as described in the section on differential gene expression. Brains from the remaining LFD and HFD fed groups were removed, frozen on dry ice and stored at −80 °C, before further processing for *in situ* hybridization.

#### Mice used in the 1 week feeding Experiment

10 male C57BL/6J mice purchased (Charles River, UK) at 7–8 weeks old and fed standard chow for 1 week before being divided into 2 weight matched groups of 5, and fed either LFD control or HFD ad libitum for 1 week with free access to water. The mice were killed by transcardial perfusion following terminal anaesthesia. Brains were immediately excised and processed for formalin fixation as previously described^52^.

### Lipopolysaccharide binding protein ELISA

LBP serum levels of WT, *Tlr4*^−/−^ and *Cd14*^−/−^ mice from the 8 week feeding experiment were assayed by Mouse LBP ELISA Kit (Enzo Life Sciences, Switzerland) according to the manufacturer’s protocol.

### Caecal contents DNA extraction of WT, *Tlr4*^−/−^ and *Cd14*^−/−^ mice

DNA was extracted from caecal contents using a FastDNA® SPIN Kit for Feces (MP Biomedicals 116570200, MP Biomedicals SARL, Illkirch, France). The samples (~100 mg) were placed in a Lysing Matrix E tube with 825 µl of Sodium Phosphate Buffer, and 275 µl of PLS solution and processed according to the manufacturer’s instructions. Eluted DNA concentration and purity was assessed using a NanoDrop ND-1000 spectrophotometer (Thermo Fisher Scientific, Wilmington, DE, USA).

### PCR amplification and Illumina MiSeq sequencing

DNA extracted from caecal samples was used as a template for PCR amplification of the V1–V2 variable regions of the bacterial 16S rRNA gene using barcoded primers MiSeq-27F 5′-AATGATACGGCGACCACCGAGATCTACACTATGGTAATTCCAGGTTYGATYMTGGCTCAG-3′ and MiSeq-338R 5′-CAAGCAGAAGACGGCATACGAGAT-barcode-AGTCAGTCAGAAGCTGCCTCCCGTAGGAGT-3′ containing adaptors for downstream Illumina MiSeq sequencing. Each sample was PCR amplified as previously described^38^.

Following amplification, the quadruplicate PCR reactions for each sample were pooled, purified by ethanol precipitation, and the DNA was resuspended in 30 μL TE. Pooled PCR amplicons were quantified using a Qubit dsDNA HS Assay Kit (Invitrogen, CA, USA, Q32854) and a Qubit 2.0 Fluorometer (Invitrogen, CA, USA) following the manufacturer’s protocol, as previously described^38^.

An equimolar mix was prepared for sequencing using equimolar concentrations of DNA from each sample. The amount of each sample to be added was calculated using the following formula: sample volume (in microliters) = DNA conc. of the sample with highest DNA conc. of all the samples (in nanograms per microliter)/DNA conc. of sample (in nanograms per microliter). All samples were above the minimum accepted concentration of 3 ng/μL. The equimolar mixed samples were cleaned using gel purification and a Wizard SV Gel and PCR Clean-Up System (Promega, A9281, Madison, WI, USA), as previously described^38^.

Paired-end sequencing of the pooled equimolar mix of PCR products was carried out on an Illumina MiSeq machine, using a read length of 2 × 250 bp. Illumina MiSeq sequencing was carried out by the Centre for Genome Enabled Biology and Medicine, University of Aberdeen. The data obtained from Illumina MiSeq sequencing were analysed using the mothur software package^53^ and based on the mothur MiSeq standard operating procedure^54^. A DNA extraction kit blank processed with only water was included as a control. The Perseus^55^ chimera removal software was used to detect and remove chimeric molecules created during PCR amplification. The Ribosomal Database Project (RDP) reference database (Release 10)^56^ was used to assign taxonomic classifications to each OTU at the phylum, family, and genus levels. Single unique sequences were not removed. Sequences classified as belonging to the *Lactococcus* genus were removed from the dataset as previously described^38^.

The final dataset contained a total of 1,321,958 sequences. One LFD *Cd14*^−/−^ sample returned only 552 sequences and was removed from the analysis. The remaining samples ranged between 9662–53849 sequences. Samples were then rarefied (sub-sampled) to reduce all sequences to 9662 to equalize the sequencing depth between all samples. A representative sequence for each OTU was generated, and the most abundant OTUs were manually curated against the BLAST database.

Sequence data have been deposited in the European Nucleotide Archive and is available under study accession number (PRJEB26713) and sample accession numbers (ERS2483599 to ERS2483645) and are detailed in Supplementary File 1. The sequence files can be found at the address: https://www.ebi.ac.uk/ena/data/view/PRJEB26713.

### Hypothalamic RNA extraction

A hypothalamic block was dissected from each mouse brain. First, frozen brains were thawed from −80 °C to −20 °C and then hypothalamic tissue weighing roughly 30 mg was dissected with coronal cuts at stereotaxic coordinates 0.38 mm and −2.28 mm relative to Bregma^57^. The hypothalamus was removed from the remaining brain tissue by making an axial cut at the top of the third ventricle and sagittal cuts at the lateral margins of the optic tracts. The frozen tissue blocks were transferred into Lysis Buffer (QIAGEN, Hilden, Germany) containing β-mercaptoethanol, and then lysed and homogenised with zirconia beads (BioSpec Products, Bartlesville, OK, USA) as recommended in the QIAGEN RNeasy Mini Kit. Total RNA was isolated from the tissue lysate using a QIAGEN RNeasy Mini Kit (QIAGEN, Hilden, Germany) with an on-column DNase digestion treatment following the manufacturer’s protocol. The RNA concentration and purity of isolated RNA was determined with a NanoDrop ND-1000 spectrophotometer (Thermo Fisher Scientific, Wilmington, DE, USA) with the spectral absorption at 260 and 280 nm. RNA integrity was assessed with an Agilent Bioanalyzer 2100 (Agilent Technologies, Santa Clara, CA, USA) and using their RNA 6000 Nano chips. RNA samples were stored at −80 °C.

### Brain sectioning and *In Situ* Hybridization

Coronal brain sections from the mouse hypothalamus included the arcuate nucleus and the paraventricular nucleus and were cut at 10 µm from the stereotaxic coordinates 0.38 mm to −2.28 mm relative to Bregma^57^ with each sequentially cut section placed on successive slides numbered through 1 to 12, before returning to slide 1 and repeating the process until each slide had 5 sections, and such that each slide had the same representation of the brain region. The process was repeated through slide numbers 12–24, Slides numbered 1–12 contained the ARC region whilst slides 12–24 contained the PVN region. Rat hypothalamic sections used for *in situ* hybridization were as previously described, cut and processed^52^.

Representative slides from each animal were hybridized with a cross-species radiolabelled rodent-specific antisense riboprobe complementary to partial fragments of agouti-related peptide (*AgRP*)^58^, suppressor of cytokine siganalling-3 (*Socs3*, used for the 8 week mouse experiment)^59^, and serpin family A member 3N (*Serpina3n*) and the corresponsing sense fragments generated from cDNA, and were either mouse-specific or mouse and hamster-specific. The *Serpina3n* probe was provided as a gift from Prof Lynda Williams (Rowett Institute, University of Aberdeen) and consisted of a 543 bp cDNA mouse specific probe. Reference sequence information, PCR primers, and cloning vectors for this probe as well others amplified from mouse cDNA including *Tlr2*, *Tlr4*, *Tlr5*, *CD14* and *Socs3* are listed in supplementary Table 2.

Template DNA for Riboprobe synthesis was amplified from isolated plasmid DNA by PCR with M13 forward and reverse primers and GoTaq hot start polymerase (Promega, UK). The antisense and sense riboprobes were then amplified using T7, T3 or Sp6 RNA polymerases (Fisher Scientific UK Ltd, Loughborough, UK) as appropriate.

Hypothalamic mRNA expression was quantified by *in situ* hybridisation using established methods^58,60,61^. Briefly, brain sections were fixed, acetylated and hybridized overnight at 58 °C with sense and antisense riboprobes labelled with 35 S (1.0–1.5 × 10^10^ dpm/L). Slides were treated with RNase A, then washed two times in 2x SCC for 5 minutes, for 10 minutes in 1x SCC, and for 10 minutes in 0.5x SCC before a final wash in 1x SSC for 30 minutes at 60 °C, then dried and apposed to autoradiographic film (BioMax MR; Kodak, USA) for between 3 to 28 days depending on the strength of the radioactive signal. Autoradiographic films were scanned at 1200 d.p.i., analysed and quantified using Image-Pro Premier software version 9.2 (Media Cybernics UK, Marlow, UK), which computes the integrated optical density of the signal relative to a 14 C autoradiographic microscale (Amersham, Pharmacia Biotech UK Ltd, Little Chalfont, UK). No signals were detected for any of the sense probes in the hypothalamus.

### PCR confirmation of *Tlr4*^−/−^ and *Cd14*^−/−^ genotype

Genotype was confirmed by PCR of DNA extracted a 10 mg sample of ileum from a subset of half of the mice of each genotype from gut ileum using QIAamp DNA Micro Kit (QIAGEN) according to the manufacturer’s protocol. Primer sequences and protocols were provided by The Jackson Laboratory.

Genotyping primers for *Cd14* mice (strain B6.129S4-Cd14tm1Frm/J) were oIMR0662: 5′-CCGCTTCCATTGCTCAGCGG-3′; oIMR1314: 5′-CCAAGTTTTAGCGCTGCGTAAC-3′; oIMR1315: 5′-GCCAGCCAAGGATACATAGCC-3′. Genotyping primers for *Tlr4* mice (strain B6.B10ScN-Tlr4lps-del/JthJ) were oIMR8365: 5′-GCAAGTTTCTATATGCATTCTC-3′; oIMR8366: 5′-CCTCCATTTCCAATAGGTAG-3′; oIMR8367: 5′-ATATGCATGATCAACACCACAG-3′; and oIMR8368: 5′-TTTCCATTGCTGCCCTATAG-3′. Standard PCR reactions were performed using GoTaq® G2 Hot Start Polymerase (Promega) and 2 μl genomic DNA through an initial 2 min at 95 °C, then 30 cycles of 95 °C for 30 sec, annealing at 67 °C for CD14 or 55 °C for TLR4 for 30 secs, 72 °C for 30 sec, followed by 72 °C for 5 min using a T100 thermal cycler (Bio-Rad, Hertfordshire, United Kingdom). Amplification was confirmed using agarose gel electrophoresis. *Cd14* primers produced a Wild-Type DNA band at 840 bp and a knockout band and 600 bp. *Tlr4* primers produced a Wild-Type band at 390 bp and a knockout band at 140 bp (data not shown).

### Quantitative PCR

Total RNA was extracted from hypothalamic tissue samples using an RNeasy® Mini Kit (QIAGEN, Crawley, UK) with on-column DNase digestion. Overall total RNA quality was assessed using an Agilent Bioanalyzer (Agilent Technologies, Santa Clara, USA) and all samples gave RIN values of >9.7. cDNA was synthesised using a high capacity cDNA kit and completed using a GeneAmp® PCR System 9700 thermal cycler (both from Applied Biosystems Foster City, CA, USA). Relative gene expression analysis was carried out in line with the Minimum Information for Publication of Quantitative Real-Time PCR Experiments (MIQE) guidelines^62^. *Gapdh* and 18S rRNA gene efficiencies were compared with the genes of interest and *Gapdh* chosen as the most appropriately matched reference gene for this study. Commercially available PCR primers were used (Qiagen, Crawley, UK) for *Gapdh* (reference # QT01658692), 18S rRNA gene (QT02448075), *Socs3* (QT02488983), *Tnf* (QT00104006), Il6 (QT00098875), *Nfκb* (QT00134421), *Il1b* (QT01048355), *Cd68* (QT00254051), *Gfap* (QT00101143) and *Emr1* (QT00099617). Real-time qPCR was performed using a 7500 Fast Real-time PCR System (Applied Biosystems) using the Quantifast SYBR green detection method (Qiagen).

### Differential gene expression by RNAseq (20 week feeding experiment)

RNA was prepared from a 3 mm diameter punch of the hypothalamus was taken from freshly dissected tissue. To prepare the hypothalamic punch, following the cull of each mouse, the brain was removed and cut coronally at approximately bregma −0.1 and −3.0. The brain slice was orientated with the caudal side face up and a 3 mm punch from the ventral aspect of the hypothalamus and centrally located was removed. Punches were transferred to a microfuge tube and immediately frozen on dry ice. Tissue was stored at −70 °C until processed. RNA was extracted using the Machery-Nagel RNA isolation kit. For RNAseq, libraries were prepared with the Illumina Truseq Stranded mRNA kit and sequenced using the high output 1 × 75 kit on the Illumina Nextgen 500 platform producing 76 bp single end reads. Up to 69 million reads were produced per sample. After quality control and alignment to mouse reference genome using HISAT2^63^, alignments and reads were counted at gene locations using featuresCounts^64^. EdgeR (version 3.16.5) was used to detect differential change in gene expression^65^ between mice on a HFD and LFD. RNAseq data have been deposited in the ArrayExpress database (http://www.ebi.ac.uk/arrayexpress) under accession number E-MTAB-6929.

### Immunofluorescence

Formalin-fixed hypothalamic sections (30 μm) were washed in phosphate buffered saline (PBS) and blocked in 4% Donkey serum/0.25% Triton X-100 in PBS for 1 hr at room temperature. Tissues were then incubated overnight in 1% Donkey serum/0.25% Triton X-100/PBS solution containing the primary antibodies: goat anti-SerpinA3N (1:100, R&D System cat. no. AF4709) or rabbit anti-GFAP (1:500, Chemicon cat. no. AB5804). The next day, sections were washed in PBS and then incubated for 2.5 hr at room temperature in 1% Donkey serum/0.25% Triton X-100/PBS solution containing the secondary antibody: anti-goat-AF594 (1:500, Abcam cat. no. ab150132) or anti-rabbit-AF488 (1:500, Abcam cat. no. ab150073). After washing, tissues were mounted on Superfrost slides and coverslipped using Mowiol. Images were acquired by using an AXIOSKOP II microscope (Zeiss, Germany) and neuroanatomical landmarks identified by using the Franklin & Paxinos Mouse Brain Atlas (Elsevier).

### Statistical analysis

For microbiota sequence data Metastats (White *et al*., 2009), a non-parametric T-test, incorporating Fisher’s exact test and the false discovery rate (FDR) was used to determine whether the proportional abundance of OTUs, or phylum, were significantly different between LFD and HFD groups and between mouse genotypes. P values generated using Metastats were corrected with the Benjamini-Hochberg method (Benjamini & Hochberg, 1995) to correct for the false discovery rate across multiple comparisons. P values of p < 0.05 were considered significant. The observed richness (Sobs), the estimated (Chao1) total richness, the Shannon diversity index, and the inverse Simpson diversity index were used to calculate the bacterial diversity within each sample in the mothur software package^53^.

For qPCR data, group-wise comparisons of gene expression ratios were performed using the public domain program Relative Expression Software Tool (REST; http://www.gene-quantification.info/)^66^. The gene expression values were normalised to the reference gene Gapdh and the data are presented as median expression levels relative to the level in the LFD group.

Unless otherwise stated group comparisons for all data were performed using R version 3.5.0 with RStudio Version 1.1.443. The normal distribution of sample data being analysed was initially tested using the Shapiro-Wilk test. Sample groups that failed the Shapiro-Wilk test were plotted and visually assessed for normality. Comparisons between normally distributed samples were analysed by One Way ANOVA followed by Tukey’s post-hoc test for multiple comparisons. Comparisons between skewed samples were analysed using Kruskal-Wallis One Way ANOVA on Ranks followed by Dunn’s test for multiple comparisons. P values of p < 0.05 were considered significant.

## Electronic supplementary material

Supplemental tables and figures

Supplementary dataset
