# Supplementary material for: Diet induced obesity is independent of metabolic endotoxemia and TLR4 signalling, but markedly increases hypothalamic expression of the acute phase protein, SerpinA3N
Source: Sci Rep. 2018 Oct 23;8:15648. doi: 10.1038/s41598-018-33928-4 (PMC6199263; doi:10.1038/s41598-018-33928-4)
Supplement: Supplementary file 1 — Supplemental tables and figures [file 41598_2018_33928_MOESM1_ESM.pdf]

Diet induced obesity is independent of metabolic endotoxemia and *Tlr4* signalling, but markedly increases hypothalamic expression of the acute phase protein, SerpinA3N.

Matthew J. Dalby, Gabriella Aviello, Alexander W. Ross, Alan W. Walker, Perry Barrett, and Peter J. Morgan

Supplementary figures

Supplementary Table 1. Nutritional composition of the LFD and HFD.

Diet	Control diet		High-Fat	
Diet code	D12450J		D12492	
	gram (%)	kcal (%)	gram (%)	kcal (%)
Protein	19.2	20	26.2	20
Carbohydrate	67.3	70	26.3	20
Fat	4.3	10	34.9	60
Total		100		100
Kilocalories/gram MJ/kg	3.85		4.4	
Ingredient	gram	kcal	gram	kcal
Casein, 80 Mech	200	800	200	800
L-Cystine	3	12	3	12
Corn Starch	506.2	2024.8	0	0
Maltodextrin 10	125	500	125	500
Sucrose	68.8	275.2	68.8	275.2
Cellulose, BW200	50	0	50	0
Total fibre	50	0	50	0
Soybean oil	25	225	25	225
Lard	20	180	245	2205
Mineral Mix S10026	10	0	10	0
DiCalcium Phosphate	13	0	13	0
Calcium Carbonate	5.5	0	5.5	0
Potassium Citrate, 1 H2O	16.5	0	16.5	0
Vitamin mix, V10001	10	40	10	40
Choline bitartrate	2	0	2	0
FD&C Yellow Dye #5	0.04	0	0	0
FD&C Blue Dye #1	0.01	0	0.05	0
Total	1055.05	4057	773.85	4057

Supplementary Table 2: PCR Primers used for application of probes for in situ hybridisation

Gene/ probe length	NCBI reference	Forward	Reverse	Cloning vector
Serpina3n **543bp**	NM_009252.2	CTACGCGGGCAAGAGGA	AAGGGGGCAATTTCAGTTT	PCR Script
Tlr2 **2185bp**	NM_011905.3	GGCCGCTCCAGGTCTTTCAC	AGGGCCACTCCAGGTAGGTCTTG	Zero Blunt TOPO PCR
Tlr4 **2464bp**	NM_021297.3	ATCATGGCACTGTTCTTCTCCTG	TTGCCGTTTCTTGTTCTTCCTC	Zero Blunt TOPO PCR
Tlr5 **2413bp**	NM_016928.3	GTTCCCCCACGCACCACACTT	TCCCACCACCACCACGATGAG	Zero Blunt TOPO PCR
CD14 **897bp**	NM_009841.3	GTTTGGGGGCGGCAGATGTGG	ATGGAGCTCCGGCGGTGACTACG	Zero Blunt TOPO PCR
Socs3* **510bp**	NM_007707	CCCGCGGGCACCTTTCTTATC	CCCCTCTGACCCTTTTGCTCCTTA	StrataClone Blunt PCR

PCR Script and StrataClone Blunt PCR vectors (Agilent Technologies LDA UK Limited, Stockport, Cheshire, UK). Zero Blunt TOPO PCR vector (Fisher Scientific UK Ltd, Loughborough, UK). * used for in situ hybridization of 20 weeks mouse study.

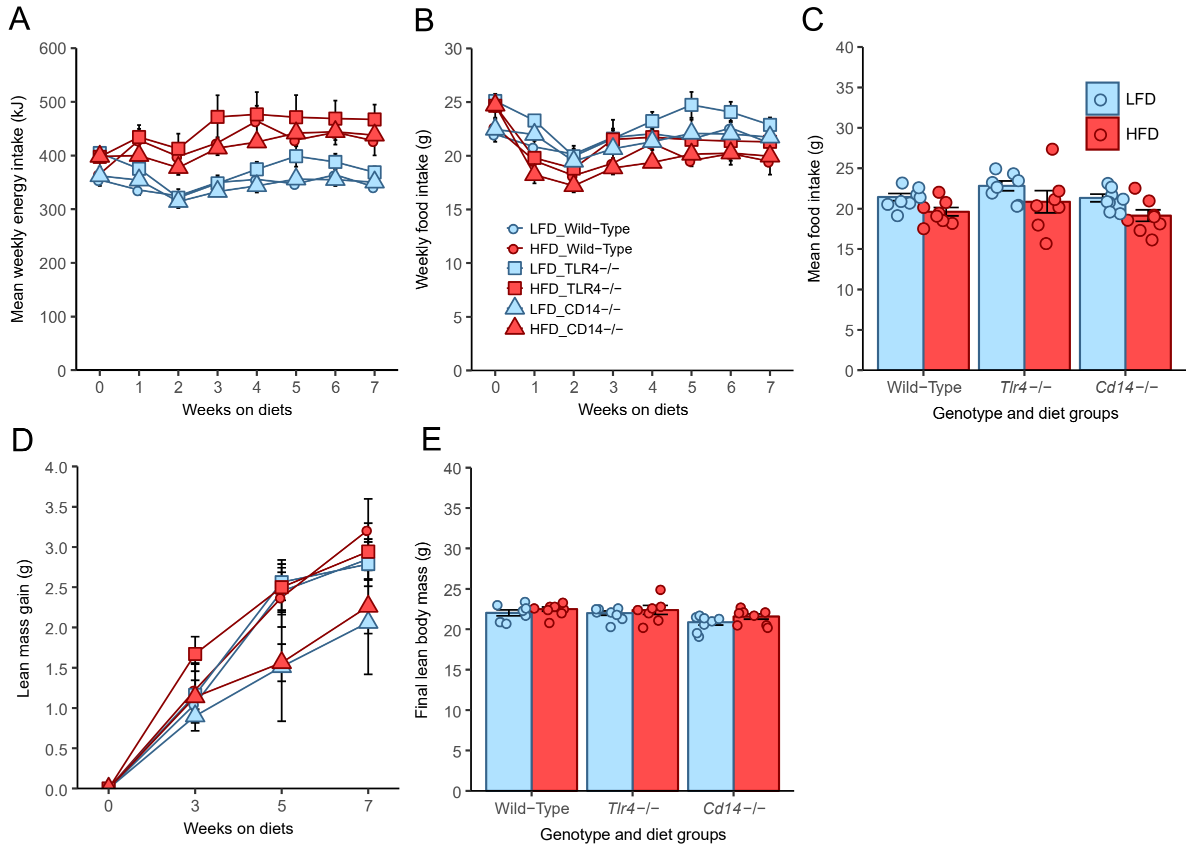

Supplementary Figure 1. Effects of diet and genotype on food intake, energy intake, and lean mass. (A) Weekly kilojoule intake; (B) Weekly food intake; (C) Mean food intake; (D) Lean mass gain; (E) Final body mass; Data represent mean ± SEM. n = 8 mice/group (n = 7 for HFD *Tlr4*$^{-/-}$ mice), circles indicate individual mice.

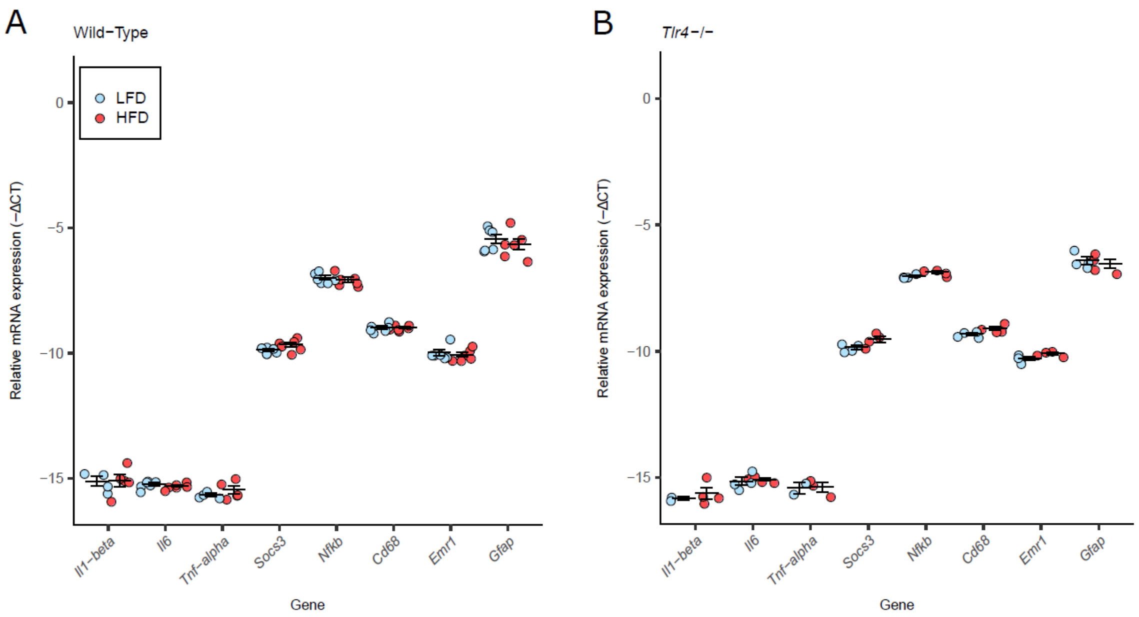

Supplementary Figure 2. Normalised delta CT values of hypothalamic genes using quantitative PCR. (A) Hypothalamic gene expression in Wild-Type mice; (B) Hypothalamic gene expression in *Tlr4*$^{-/-}$ mice. Values are expressed as the mean−ΔCt ± standard error. n = 6 mice/group for Wild-Type mice and n = 4 for *Tlr4*$^{-/-}$ mice, circles indicate individual mice.

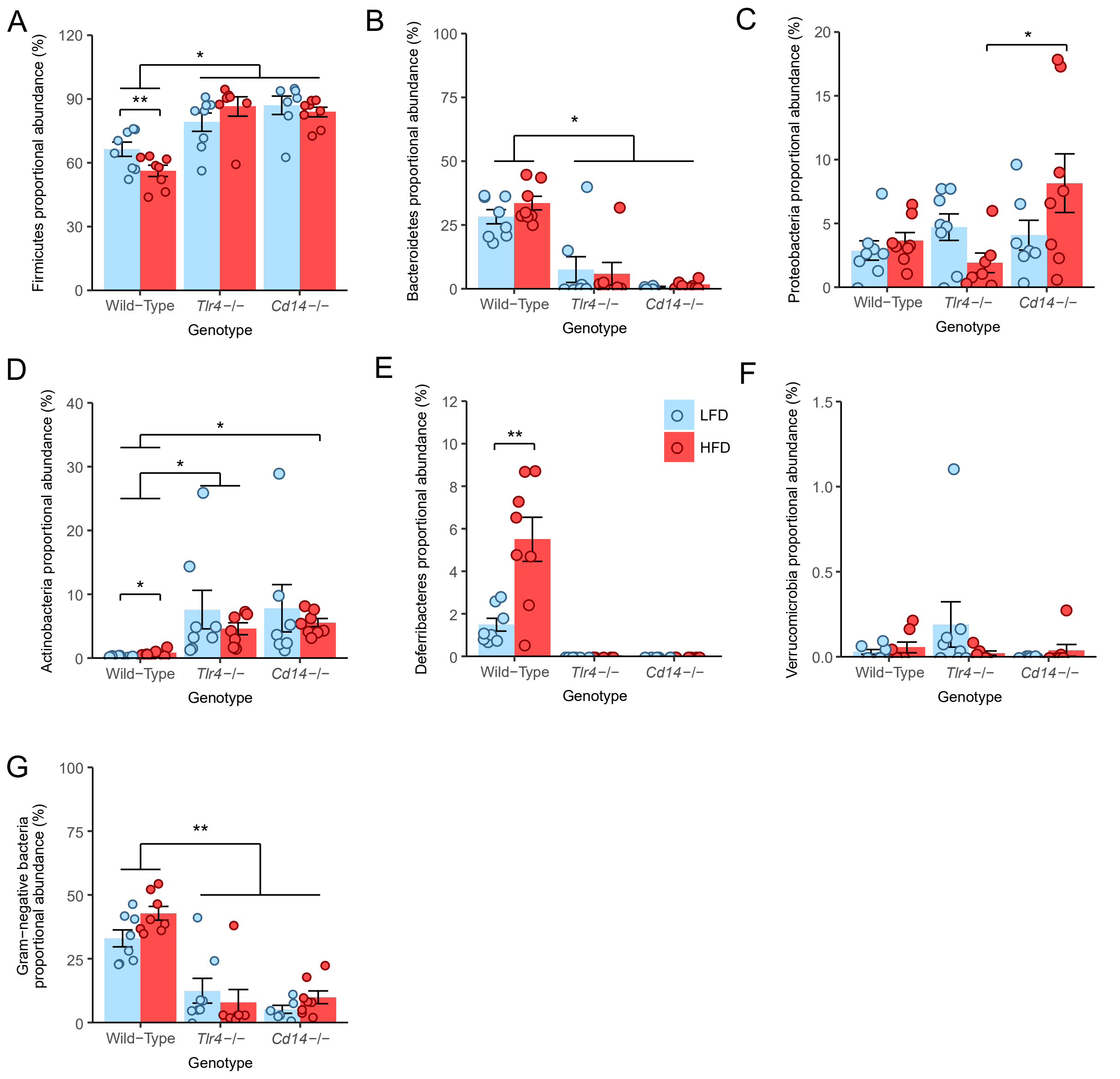

Supplementary Figure 3. Phylum level proportional abundance. (A) Proportional abundance of the Firmicutes phylum, (B) the Bacteroidetes phylum, (C) the Proteobacteria phylum, (D) the Actinobacteria phylum, (E) the Deferribacteres phylum, and (F) the Verrucomicrobia phylum. (G) Proportional abundance of Gram-negative bacteria. Data represent mean ± SEM. ∗p < 0.05, ∗∗p < 0.01, ∗∗∗p < 0.001; n = 8 mice/group (n = 7 for LFD *Cd14*$^{-/-}$ mice and HFD *Tlr4*$^{-/-}$ mice), circles indicate individual mice.

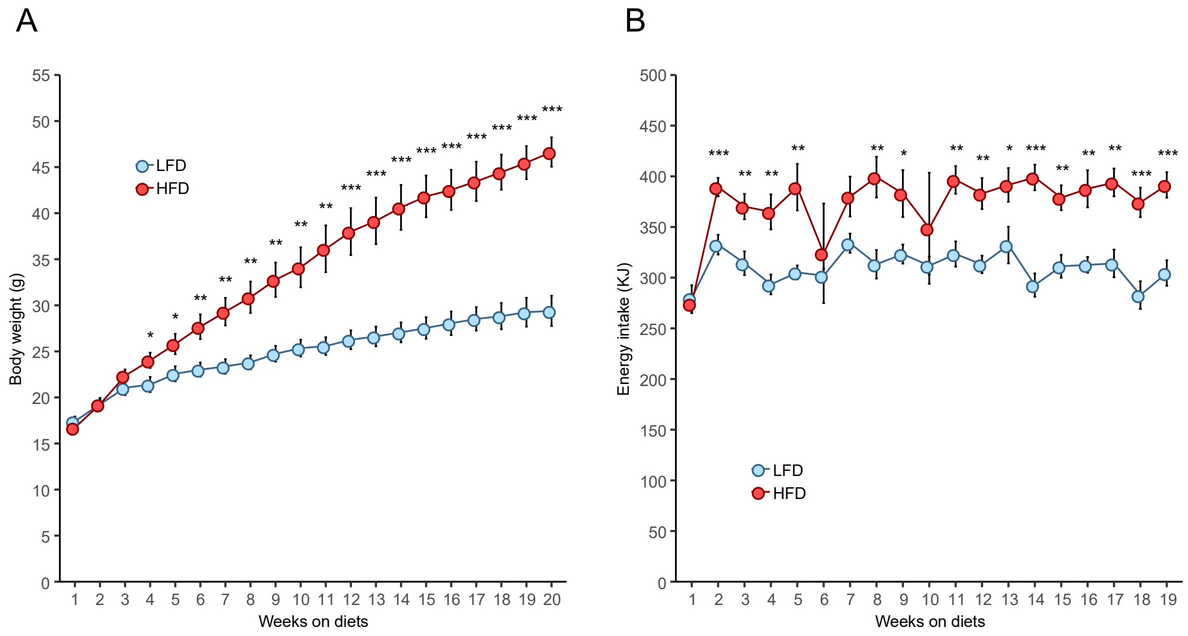

Supplementary Figure 4: Body weight and energy intake of wild-type mice fed LFD or HFD for 20 weeks. (A) Weekly mean body weight; (B) Weekly mean energy intake. Data represent mean ± SEM. ∗p < 0.05, ∗∗p < 0.01, ∗∗∗p < 0.001; n = 8 mice/group.

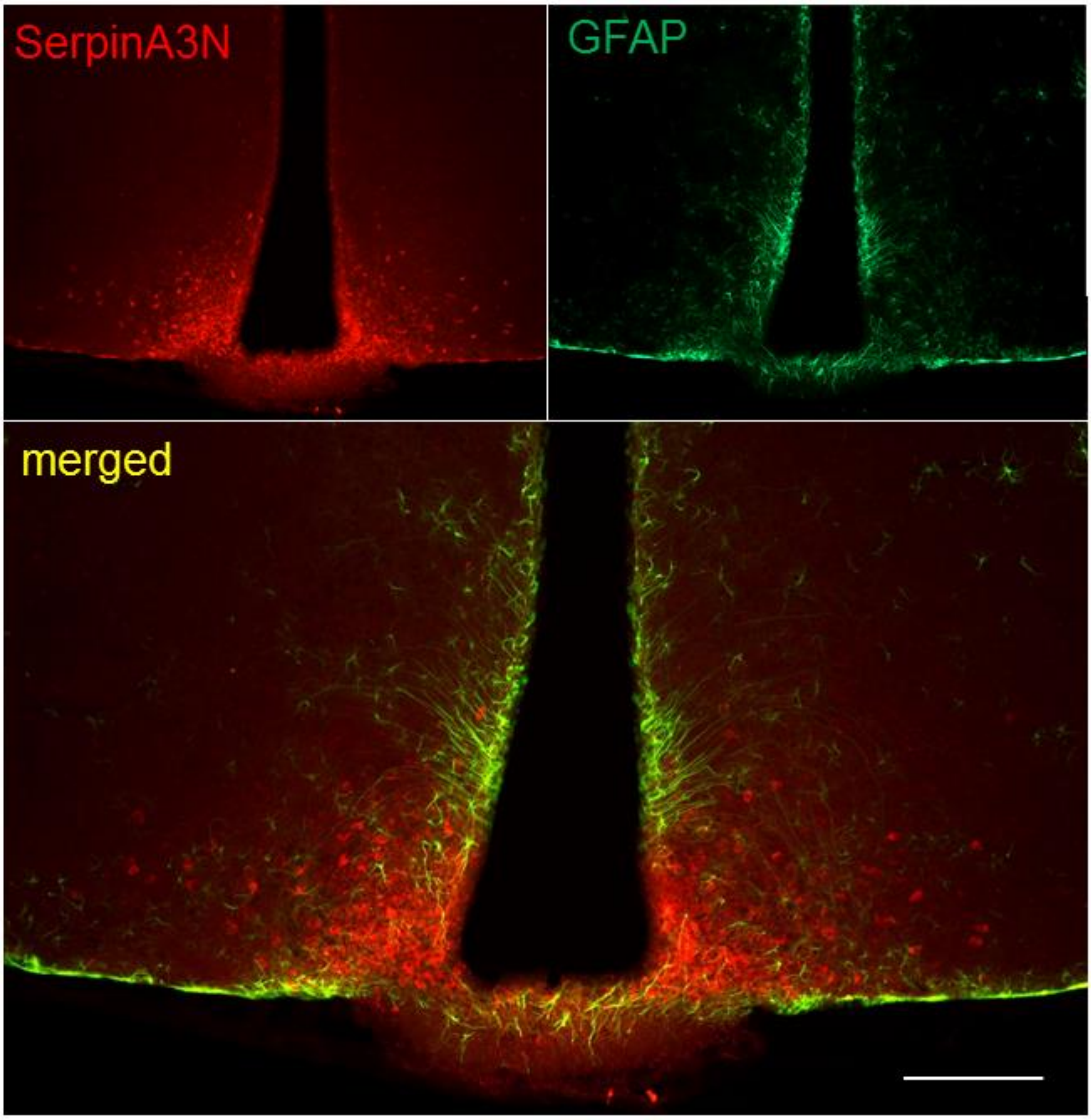

Supplementary Figure 5. SerpinA3N is not expressed by astrocytes in the hypothalamus. Representative images of LFD-fed Wild-Type mice showing absence of overlapping SerpinA3N (AF-594 conjugated) and GFAP (AF-488 conjugated) expression in the ARC. GFAP: glial fibrillary acidic protein. Scale bar = 100 μm.
